# Plasma-Induced Phase Transformation of SnS_2_ to SnS

**DOI:** 10.1038/s41598-018-28323-y

**Published:** 2018-07-06

**Authors:** Jung Ho Kim, Seok Joon Yun, Hyun Seok Lee, Jiong Zhao, Houcine Bouzid, Young Hee Lee

**Affiliations:** 1 Center for Integrated Nanostructure Physics, Institute for Basic Science (IBS), Suwon, 16419 Republic of Korea; 2 Department of Energy Science, Sungkyunkwan University, Suwon, 16419 Republic of Korea; 3 Department of Physics, Sungkyunkwan University, Suwon, 16419 Republic of Korea; 4 Department of Physics, Chungbuk National University, Cheongju, 28644 Republic of Korea; 5 Applied Physics Department, The Hong Kong Polytechnic University, Hung Hom, Hong Kong China

## Abstract

Layered van der Waals materials have recently attracted attention owing to their exceptional electrical and optical properties in thin layer form. One way to extend their utility is to form a heterostructure which combines various properties of layered materials to reveal intriguing behavior. Conventional heterostructure synthesis methods are difficult to develop and the heterostructure formed can be limited to a small area. Here, we investigate the phase transformation of SnS_2_ to SnS by removing sulfur atoms at the top surface using Ar plasma. By varying the plasma power and exposure time, we observed that SnS is subsequently formed on top of the mogul-like structure of SnS_2_. Since SnS is a *p-*type semiconductor and SnS_2_ is an *n-*type semiconductor, we naturally formed a vertical *p-n* junction. By using graphene at the top and bottom as transparent electrodes, a vertical *p-n* diode device is constructed. The device demonstrates good rectifying behavior and large photocurrent generation under white light. This method can be applied to large-area heterostructure synthesis using plasma via phase transformation of various metal dichalcogenides to metal monochalcogenides.

## Introduction

Motivated by graphene with extremely high electrical mobility but poor on/off current ratio, which is a serious drawback for switching device applications, other types of two-dimensional (2D) layered materials such as layered metal dichalcogenides and h-BN have emerged recently as promising candidates for devices with both high mobility and high on/off current ratio. Numerous research studies have focused on their physical, chemical, thermal, mechanical, and optical properties^1–4^. The most common sample preparation method is mechanical exfoliation. Since the size is limited to a few micrometers, which is an obstacle for some measurements and for industrial-scale manufacture, several methods of synthesizing large area samples have been developed. One typical simple method is chemical vapor deposition (CVD), which is a reasonable approach for wafer-scale fabrication with fairly good sample quality achievable^5–10^.

While layered metal dichalcogenides themselves reveal various intriguing properties, many interesting properties emerge in heterostructures, the construction of which using CVD approaches is still an exploratory area^11–15^. Although there are a few reports of CVD-grown vertical heterostructures, most researchers aim for lateral heterostructures. Lateral heterostructures composed of transition metals (Mo and W) and chalcogens (S and Se), such as MoS_2_-WSe_2_, MoS_2_-WS_2_, WS_2_-WSe_2_, and MoS_2_-MoSe_2_ have been grown using a CVD method^16–19^. Although the size is still limited to several micrometers, these monolayer lateral heterostructures demonstrate two well-defined regions which show typical diode behavior due to work function difference^20–22^. An atomically sharp interface is, however, difficult to control while avoiding overlapped regions. In addition, the junction area is too narrow and hence, large optical gain cannot be expected. To overcome this obstacle, CVD-grown vertical heterostructures could be an ideal platform for practical optoelectronics. When two different materials are grown vertically, the entire overlapped region can be considered as a heterojunction. The effective area for carrier transport and light detection is larger than that of a lateral heterostructure^21,22^. Furthermore, *in situ* CVD-grown vertical heterostructures may ensure a clean interface. The interface can significantly alter carrier transport and optoelectronic properties depending on the quality of interface^23,24^. Nevertheless, the realization of CVD-grown vertical heterostructures over large areas has been sparse and remains elusive.

Another approach to form heterostructures is to take advantage of structural phase transformation^25,26^. Phase transition from semiconducting to metallic phase has been provoked via laser irradiation^27,28^ or Li-intercalation^29^ to reduce contact resistance in field-effect transistors. Up to now, these methods have been restricted to the modification of phases in the lateral direction. Another interesting method to achieve vertical heterostructures is to use electron beam or laser irradiation on a SnS_2_ (tin disulfide) thin film and to convert the top layers into SnS (tin monosulfide) to form a SnS_2_-SnS heterostructure^30,31^. While this method yields an *in situ* vertical heterostructure at the irradiated area, which is advantageous for micro-patterning, phase conversion of the whole area is still not easily achievable.

In this work, we used Ar ion bombardment to induce the phase transformation of a SnS thin film and constructed a semiconductor heterostructure of the converted top SnS and the remaining bottom SnS_2_. Sulfur atoms are partially kicked out from SnS_2_ to form a stable orthorhombic SnS phase. The constructed vertical heterostructure of *n*-SnS/*p*-SnS_2_ forms a type II *p-n* band alignment, which clearly demonstrates the diode characteristics^32,33^. This method is applicable for phase engineering over large areas.

## Methods

### Sample preparation

Commercially available SnS_2_ single crystal was purchased (2D semiconductors). The SnS_2_ crystal was then transferred onto a 300 nm SiO_2_ (300 nm)/Si substrate by mechanical cleavage. Ar plasma treatment was conducted by a reactive ion etch (RIE) system (AFS-R4T, All For System). The system is equipped with CCP (capacitively-coupled plasma) source type bottom plasma source with a 13.56 MHz bottom RF generator. Prior to plasma exposure, the vacuum chamber was filled with Ar gas of 50 sccm for 10 minutes to remove any residual gas such as N_2_ or O_2_. The as-prepared sample was put into the RIE vacuum chamber and pumped out to reach a basal pressure below 20 mtorr. Ar gas flow was fixed to 20 sccm for all experiments. The power of Ar plasma was controlled from 40 W to 140 W depending on the experimental conditions. Exposure time was also varied from 20 s to 120 s. An optical microscope (BX51RF, Olympus) was used to observe color and morphology change before and after plasma treatment. All procedures mentioned above were conducted in a clean room of a class of 1000.

### Characterization

Thickness and morphology were checked using atomic force microscopy (AFM) (E-sweep, Seiko). Micro-Raman spectroscopy (RM1000-Invia, Renishaw, 532 nm) were performed before and after the plasma treatment for material property analysis. For transmission electron microscopy (TEM) measurements (JEM ARM200F, JEOL), two types of samples were prepared. For top-view measurement, a SiO_2_/Si substrate with plasma-treated flakes was immersed in a buffered oxide etchant (1178-03, J.T. Baker) for detachment. The resulting flakes were transferred onto a TEM grid. To obtain cross-sectional TEM images, the samples were prepared with a focused ion beam (JIB-4601F, JEOL). For elemental analysis, Energy-dispersive X-ray spectroscopy (EDS) in TEM was employed.

### Device fabrication and electrical measurement

Graphene was mechanically exfoliated on a pre-patterned SiO_2_ (300 nm)/Si substrate. The plasma-treated flake was then transferred with alignment onto graphene flake. Another piece of graphene flake was again transferred on the pre-stacked heterostructure for the top electrode. E-beam lithography was conducted to pattern electrical pads that connect from the top and bottom graphene, followed by e-beam deposition of Cr/Au (5/50 nm). Electrical characterization and photocurrent measurements were conducted with a laboratory-built probe station equipped with Agilent B2902A precision source/measure unit.

## Results and Discussions

**Figure 1:**
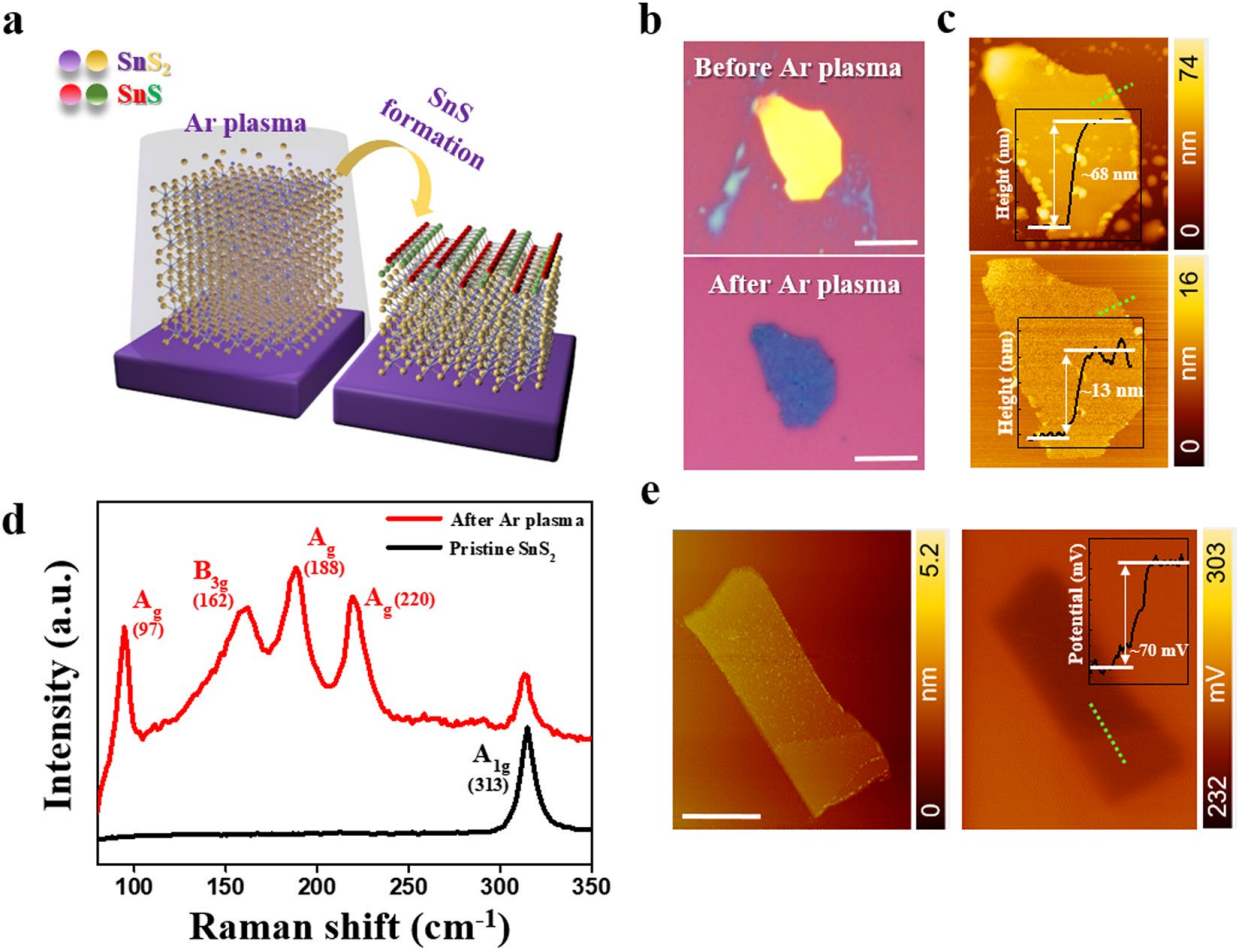
(**a**) Schematic illustration of Ar plasma treatment on exfoliated SnS_2_ crystal. (**b**,**c**) Optical image and atomic force microscopy (AFM) topography of exfoliated SnS_2_ flake before and after Ar plasma treatment. Thickness of the flake measured from green dotted line decreased from 68 nm to 13 nm. Scale bar is 10 μm. (**d**) Raman spectroscopy of before (black) and after (red) plasma treatment. New peaks relevant to SnS evolved after plasma treatment while A_1g_ peak of SnS_2_ coexist. (**e**) Kelvin probe force microscopy (KFM) scanning image of plasma treated (upper region)/pristine SnS_2_ (lower region) flake. Left figure is topography image and right figure is KFM scanned image. Scale bar is 1 μm.

A schematic illustration of SnS_2_ to SnS phase transformation is shown in Fig. 1(a). Bulk SnS_2_ was mechanically exfoliated on a Si substrate with a 300 nm thermally oxidized SiO_2_ layer. The sample was subsequently put into a reactive ion etch (RIE) chamber for plasma treatment (See Methods section). Ar plasma generated by RF plasma was accelerated to collide onto SnS_2_ flakes. After Ar^+^ radicals strike the surface of SnS_2_, they transfer their kinetic energy to the SnS_2_ flake. The transferred kinetic energy can break the Sn-S bonding. As a consequence, both S and Sn atoms can be kicked out from the SnS_2_ surface^30^. S atoms are more likely to escape from SnS_2_ than Sn atoms due to their lighter mass, which could induce a SnS phase on the top of SnS_2_. Plasma treatment will first remove SnS_2_ layers which results in thinning of the flake. Figure 1(b,c) are optical images and atomic force microscopy (AFM) images of the pristine and Ar plasma-treated SnS_2_ flake. It clearly shows that the initial color of SnS_2_ flake is yellow and altered to dark-blue, which implies a change in thickness. To confirm the thickness, we conducted an AFM profile across the boundary indicated by the green dotted line. The inset in Fig. 1(c) clearly shows that the thickness decreases from 68 nm to 13 nm after plasma treatment.

Figure 1(d) is the Raman spectrum of an Ar plasma-treated SnS_2_ sample with pristine SnS_2_ for comparison. Only one Raman mode (A_1g_) near 313 cm^−1^ appeared in the pristine SnS_2_ sample^33,34^. However, new peaks corresponding to Raman modes of SnS of ~97, ~162, ~188 and ~220 cm^−1^^33,35^, emerged in the plasma-treated sample. Meanwhile, the A_1g_ peak of SnS_2_ sample was still present. This implies a partial phase transformation of SnS at the top layers and the remaining SnS_2_ at the bottom layers. To demonstrate the phase transformation, we used Kelvin probe force microscopy (KFM) scanning. For this sample, we deposited Au metal to partially passivate the surface of the SnS_2_ flake via patterning by electron beam lithography. We then exposed the sample to plasma irradiation. The Au layer was etched away later by Au etchant (TFA, Transene). The left image is a topography scanning image which shows two distinct regions with different contrast. The upper region is the plasma-treated region while the lower region is a pristine region. Interestingly, the KFM shows a distinct contrast, with a chemical potential difference of ~70 mV (right in Fig. 1(e)), which is attributed to the emerging phase of SnS at the exposed area.

**Figure 2:**
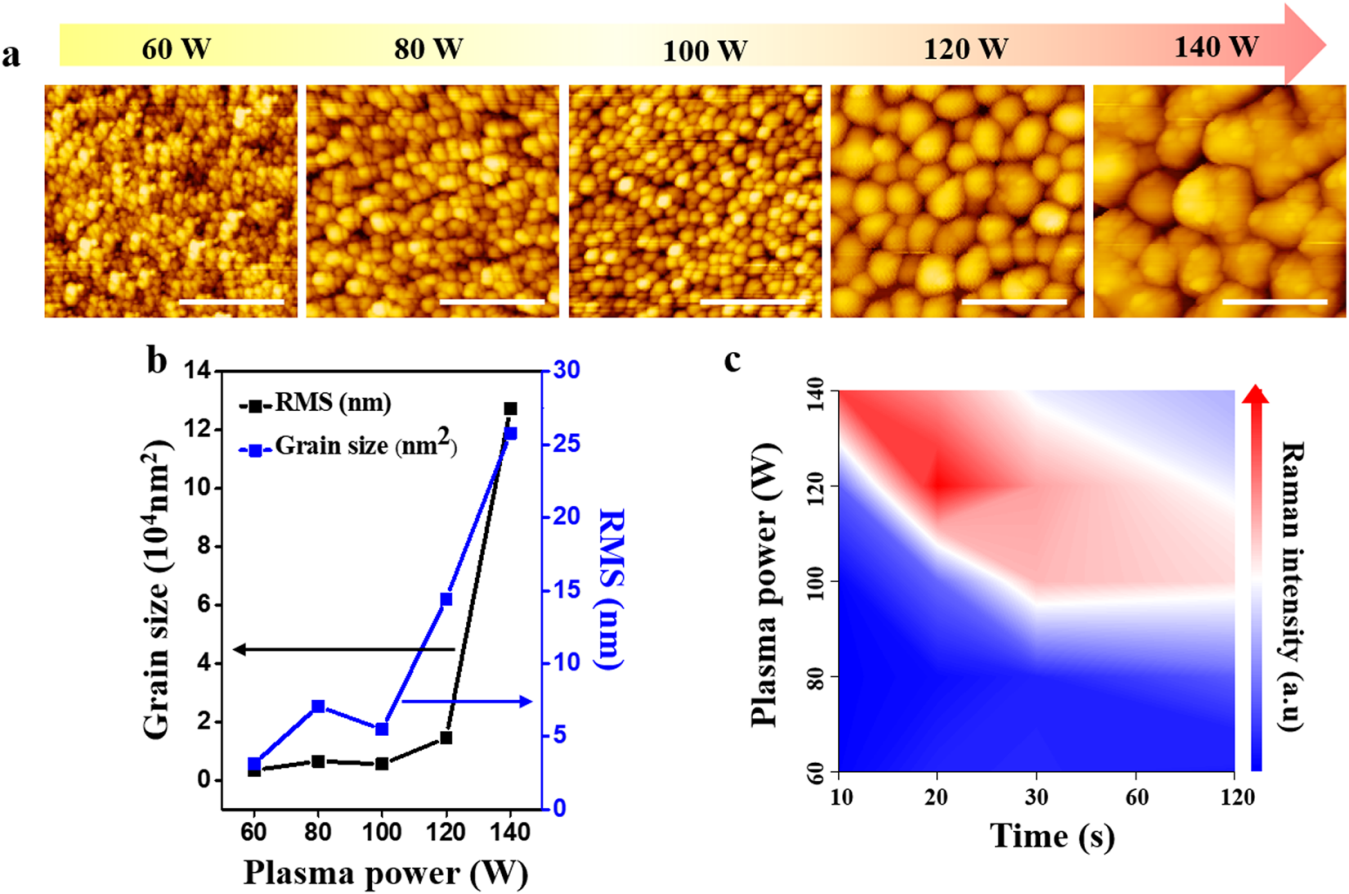
(**a**) AFM scanning of plasma treated surface of SnS_2_ flake. All images were exposed for 20 seconds. Scale bar insist 500 nm. (**b**) Grain size and root-mean-square (RMS) value variation under different plasma power. Grain size and RMS value increase significantly as plasma power increases. (**c**) SnS peak evolution measured by Raman spectroscopy with varied time and plasma power. The graph is mapped according to SnS A_g_ peak (97 cm^−1^).

To observe the evolution of phase and morphology change, we conducted AFM measurements. Figure 2(a) shows the AFM topography of the plasma-treated SnS_2_ flake. The plasma power was varied from 60 W to 140 W, while fixing Ar gas flow at 20 sccm and exposure time to 20 s. We clearly observe granular structures on the sample surface. Unlike ideal isotropic etching, the surface of the flake has mogul-like shapes after plasma treatment. As the power increases, the hemispheres of the moguls grow larger in diameter and the root-mean-square (RMS) value also increases (Fig. 2(b)).

We investigated the evolution of phase change from SnS_2_ to SnS as a function of the power and time for plasma. The plasma power was varied from 60 W to 140 W in intervals of 20 W, while the exposure time was chosen to be 10, 20, 30, 60, and 120 s. Ar gas flow was fixed at 20 sccm. Figure 2(c) shows the intensity changes of the SnS A_g_ peak near 97 cm^−1^, a typical Raman peak that implies phase transformation from SnS_2_ to SnS. The peak intensity is at a maximum at around 120 W for 20 s. At low plasma power, no SnS peak was detected, even at a longer exposure time of 120 s. The SnS_2_ surface was etched away, resulting in thinning of SnS_2_ with minimal SnS formation. Excessive plasma treatment with high power and long exposure time decreases the SnS peak, presenting degradation of the SnS lattice. We conclude that an intensive exposure at high power and short time would maximize the amount of SnS on the SnS_2_ surface.

**Figure 3:**
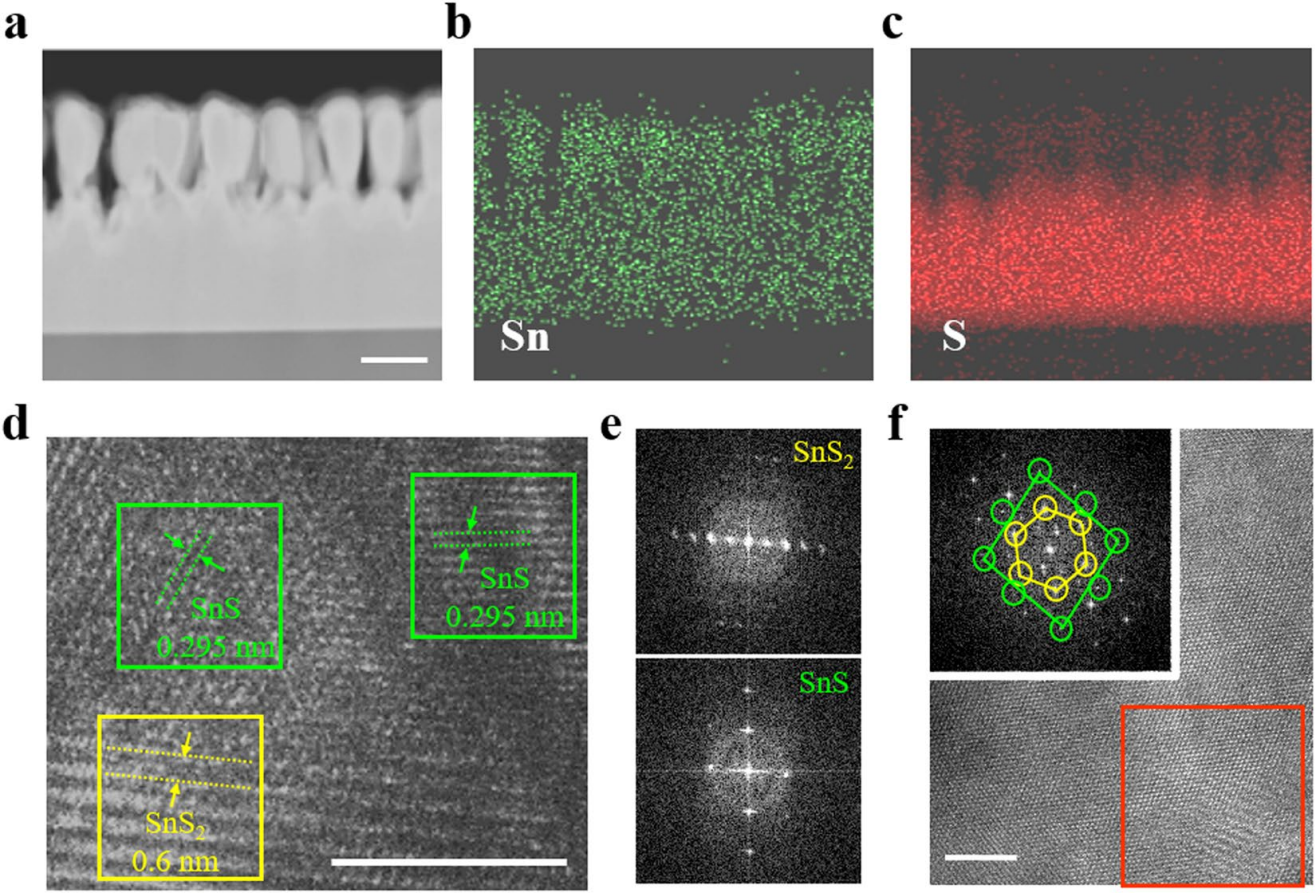
(**a**) Cross-section transmission electron microscopy (TEM) image of plasma treated flake. Plasma was exposed under 120 W for 180 seconds. Scale bar is 100 nm. (**b**,**c**) EDS mapping of the same area. Sn exist throughout the flake whereas S ratio decrease where plasma was exposed. (**d**) HR-TEM cross-section image of boundary region between SnS_2_ and SnS. The difference between two materials can be identified by significant change of interlayer distance. Scale bar is 2 nm. (**e**) Fast Fourier transform (FFT)-diffraction analysis of each material. (**f**) Top-view HR-TEM image. FFT-diffraction pattern is obtained from red boxed region. It shows coexistence of both SnS (orthorhombic) and SnS_2_ (hexagonal). Scale bar is 10 nm.

To analyze its atomic structure, cross-sectional transmission electron microscopy (TEM) was used. The flake was exposed to 120 W Ar plasma for 180 s and subsequently cut with a focused ion beam (FIB) for cross-section imaging. Figure 3(a) shows a cross-section image which reveals pillar-like structures formed on the upper part of the flake. Although the exact mechanism is not clear, it is possible that plasma preferentially attacks defects under optimum plasma conditions, resulting in a different etching rate in the perpendicular direction, thus creating a pillar-like structure. Meanwhile, plasma removes sulfur atoms from the remaining SnS_2_. This leads to SnS formation throughout the surface of the pillar structure, increasing the SnS portion, as evidenced by the Raman signals. Energy-dispersive X-ray spectroscopy (EDS) mapping of the same region indicates that while Sn atoms exist in the overall region (Fig. 3(b)), the S ratio was dramatically reduced at the top of the pillar structure (Fig. 3(c)). Figure 3(d) is a cross-sectional high-resolution transmission electron microscopy (HR-TEM) image of the pillar-like region. The configuration depicts vertical heterostructure overall but reveals a mixed orientation of SnS on single-crystal SnS_2_. As shown in Fig. 3(d), two distinct regions can be observed with a significant difference in the interlayer distance. The pristine SnS_2_ region has an interlayer distance of 0.6 nm, while the SnS region has an interlayer distance of 0.295 nm^36–38^. Despite the fact that the SnS_2_ region has a single-crystalline structure, the plasma etching results in the formation of poly-crystalline SnS. The crystal orientation of the SnS is randomly formed above SnS_2_. Fast Fourier transform (FFT)-diffraction analysis observed in each region is shown in Fig. 3(e). The interlayer distance of two materials can be precisely measured from this image. Furthermore, it is difficult to obtain structural information from top-view HR-TEM but the FFT-diffraction image obtained from the red-boxed region shows coexistence of the two materials (Fig. 3(f)). Both hexagonal SnS_2_ and orthorhombic SnS can be observed from FFT-diffraction^33^.

**Figure 4:**
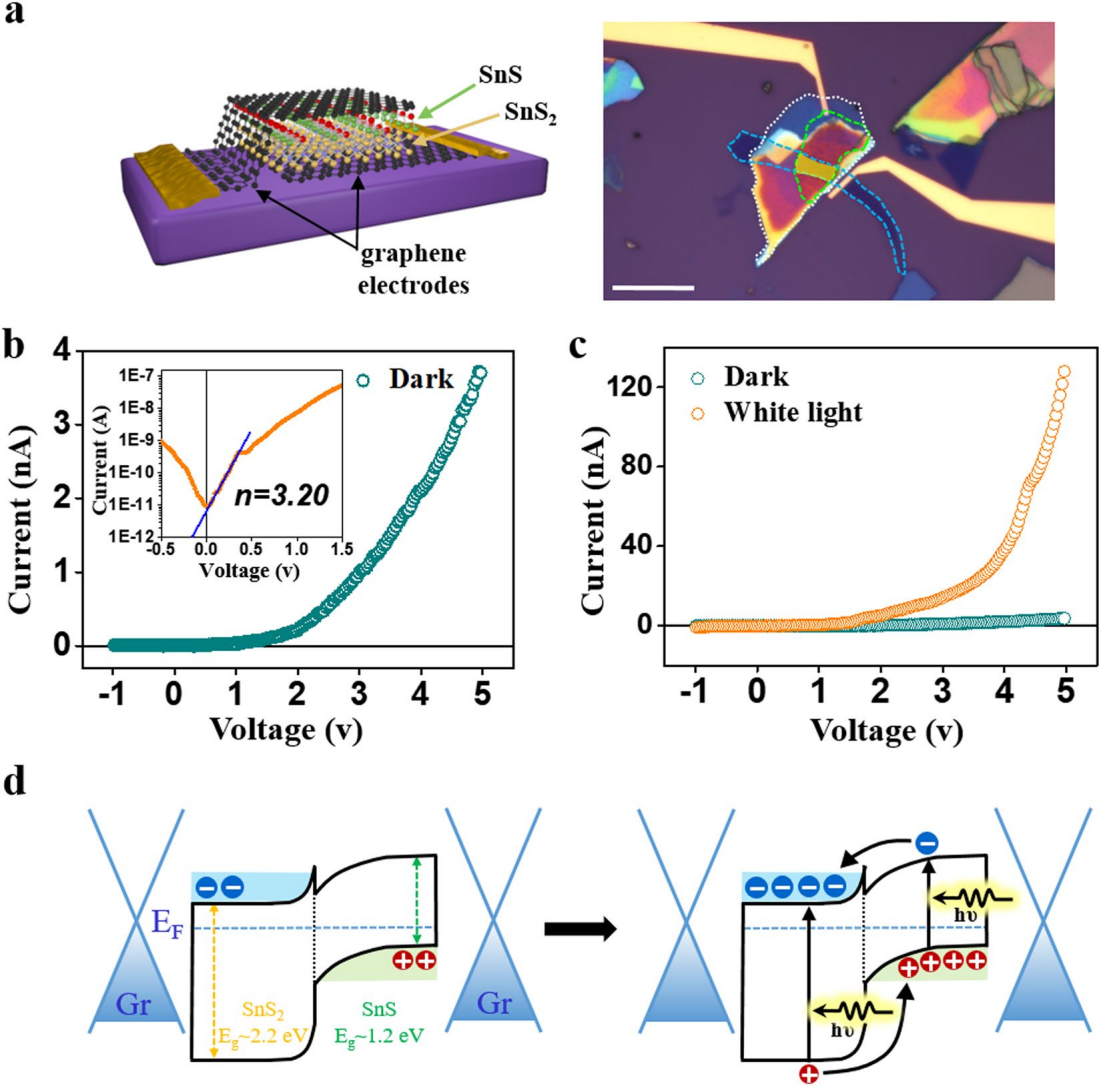
(**a**) A schematic illustration (left) and optical image (right) of SnS-SnS_2_ heterostructure *p-n* diode. The scheme illustrates graphene-SnS-SnS_2_-graphene stacked layers. Optical image shows the top-view of the device. Plasma treated SnS_2_ flake (SnS-SnS_2_ heterostructure; white dotted line) is sandwiched between bottom graphene electrode (blue dotted line) and top graphene electrode (green dotted line). Active device region (yellow colored area) is the overlapped region. Scale bar is 10 μm. (**b**) Rectifying behavior of the diode. Ideality factor (n) is calculated to be 3.20 (inset). (**c**) Photocurrent measurement under white light exposure. Under white light illumination, rapid increase of photocurrent is observed. (**d**) A schematic drawing of band alignment and photocurrent generation of graphene-SnS-SnS_2_-graphene vertical device.

We further demonstrated a vertical transistor using the SnS-SnS_2_ heterostructure. Figure 4(a) shows a schematic illustration and optical image of a SnS-SnS_2_ heterostructure vertical device. After plasma treatment, the SnS-SnS_2_ heterostructure (white dotted area) was align-transferred on the bottom graphene electrode (blue dashed area)^39^. Graphene electrodes are known to form good electrical contact with two-dimensional layered structures, accompanying optical transparency^40,41^. To perform as an optoelectronic device, we used another piece of graphene as a transparent top electrode, which allows for light penetration to the heterojunction (green-dashed area). The graphene contacts are connected to larger contact pads with Cr/Au deposition. The SnS (*p*-type) and SnS_2_ (*n*-type) form a *p-n* junction and show typical rectifying diode behavior (Fig. 4(b)). The diode performance shown in Fig. 4(b) (inset) can be estimated using the Shockley diode equation, which is expressed as:

$$I={I}_{0}(\exp (\frac{q{\rm{V}}}{n{\rm{kT}}})-1)$$

where *I* is diode current, *I*_0_ is reverse bias saturation current, *V* is the voltage across the diode, *n* is ideality factor and *T* is temperature. *q* and *k* are constants which are elementary charge and Boltzmann constant, respectively. Ideality factor (*n*) usually relates to junction quality and varies from 1 to 2 in a conventional diode^42^. From the Shockley diode equation, our device shows an ideality factor of *n* = 3.20. This high *n* value is attributed to defects and trap sites at the junction area developed during plasma treatment which promote the carrier recombination process. Previous reports from the literature have shown that in two-dimensional layered structures, an ideality factor of >2 is frequently observed since the defect sites play a more significant role^43–45^, while defect healing methods can improve such issues^46^. Photoresponse effects were qualitatively measured using white light illumination on the device. A large photocurrent, ~35 times current flow is observed at V_DS_ = 5 V (Fig. 4(c)). The mogul-like shape is advantageous for light harvesting devices such as solar cells, because such three-dimensional structures can efficiently trap incident light^47^. A possible photocurrent generation mechanism was depicted in Fig. 4(d). SnS_2_ is an *n*-type semiconductor with ~2.2 eV bandgap. SnS, on the other hand, is a *p*-type semiconductor with ~1.2 eV bandgap^33,48,49^. SnS_2_ and SnS form staggered band alignment with type II heterojunctions^33,50,51^, resulting in typical rectifying diode behavior as shown in Fig. 4(b). Typical photovoltaic photocurrent generation from *p-n* junction should reveal the open-circuit voltage (V_oc_) and short-circuit current (I_sc_). However, these parameters do not appear clearly in this measurement, which is attributed to carrier recombination from large trap density caused by plasma damage. Therefore, we explain our photoresponse results with the photoconductivity effect. Under light illumination, carriers are photo-excited in both materials creating electrons and holes. Due to band bending, electrons that are created in SnS move to lower energy region (SnS_2_ conduction band) while hole that are created in SnS_2_ move to SnS valence band. These carriers reach top and bottom graphene contacts under applied bias.

## Conclusion

We have investigated the phase transformation of SnS_2_ to SnS by Ar plasma treatment. By removing S atoms on the surface of SnS_2_, we successfully synthesized a SnS-SnS_2_ heterostructure. Anisotropic etching is observed which forms pillar-like structure on the surface which is covered with SnS. SnS is well formed when exposed to high power for a short duration. Since SnS is a *p-*type and SnS_2_ is an *n-*type, we made a vertical diode using graphene for transparent electrodes and measured IV characteristics. Electric field formation between the two materials led to photocurrent generation under white light illumination.
